# Supplementary figures and images for: Humanized Extracellular Vesicles for Efficient RNA Delivery
Source: bioRxiv. 2025 Dec 17:2025.12.15.694436. Preprint. [Version 1] doi: 10.64898/2025.12.15.694436 (PMC12724605; doi:10.64898/2025.12.15.694436)

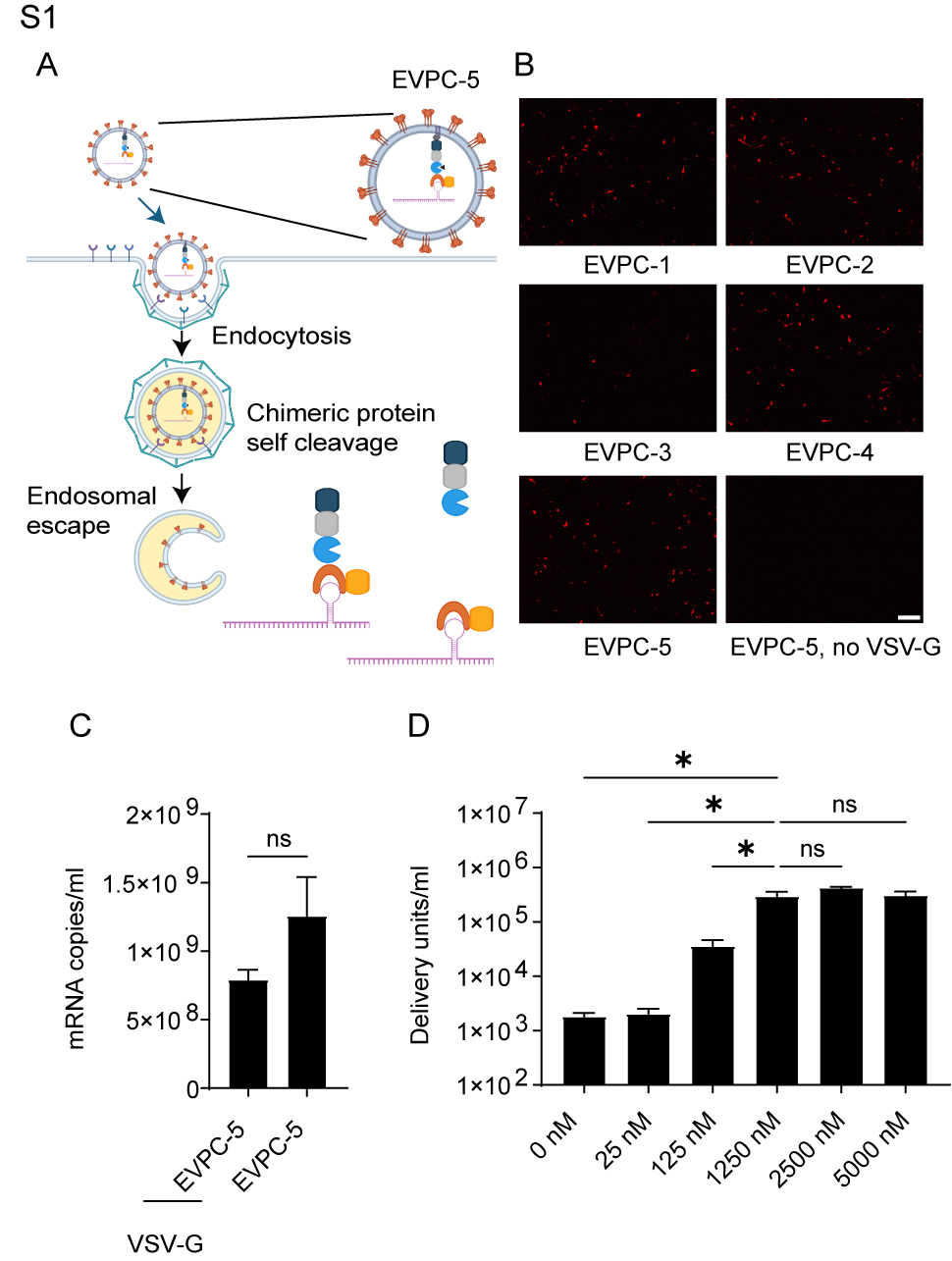

Supplement: Supplement 3 [file media-3.tif]

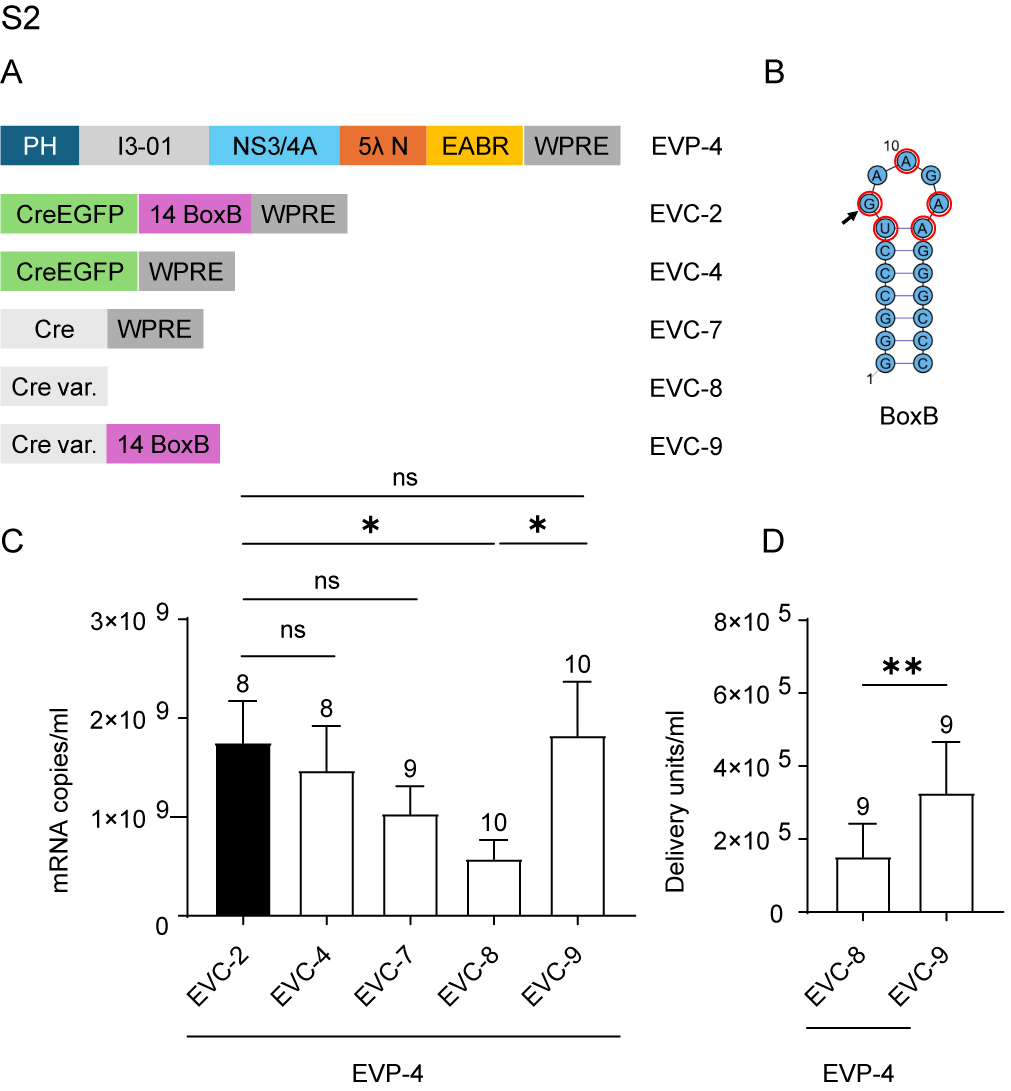

Supplement: Supplement 4 [file media-4.tif]

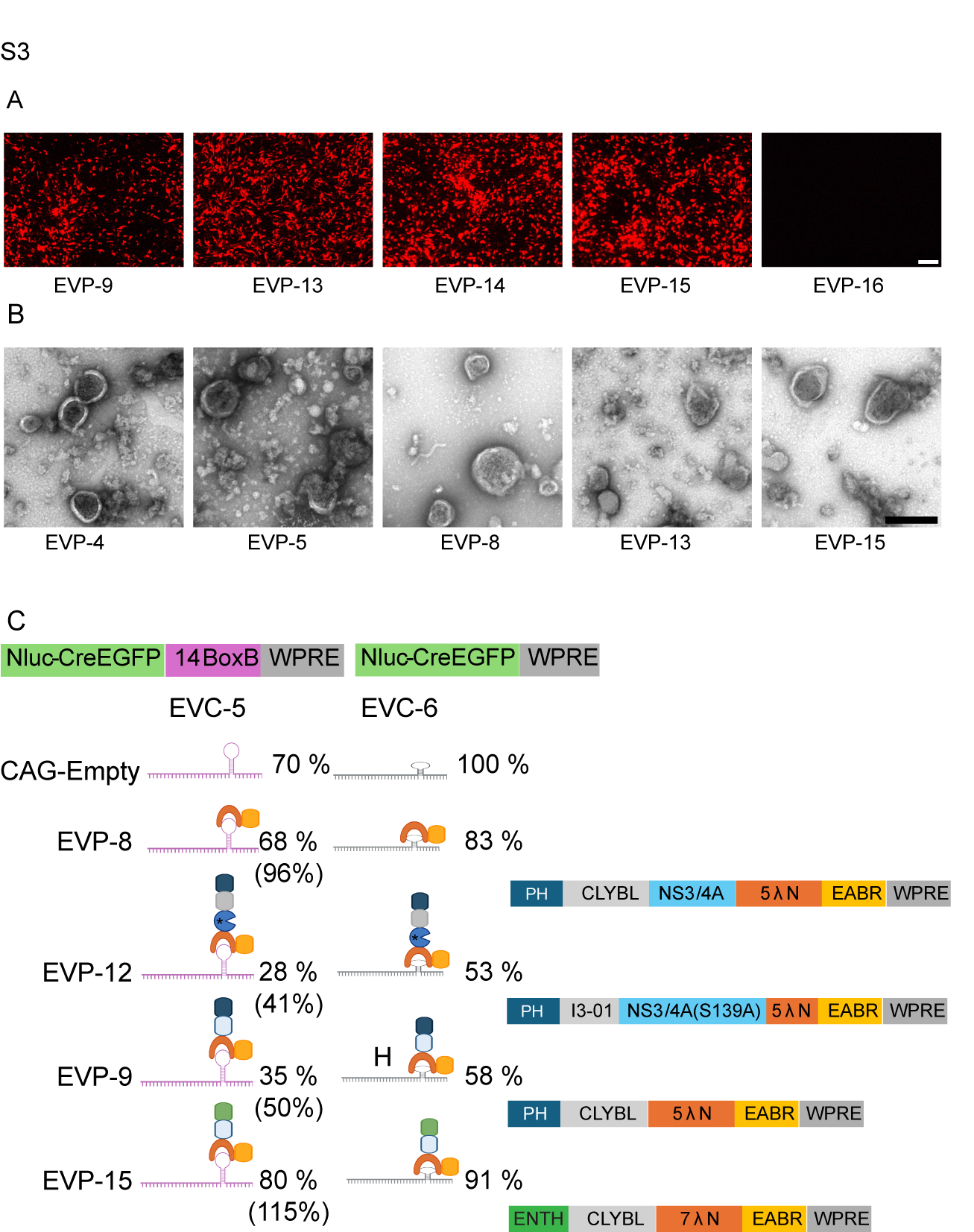

Supplement: Supplement 5 [file media-5.tif]
